# Measurement of urinary pesticide biomarkers among Latina farmworkers in southwestern Idaho

**DOI:** 10.1038/s41370-020-00285-2

**Published:** 2021-01-20

**Authors:** Cynthia L. Curl, Lisa Meierotto, Rebecca L. Som Castellano, Meredith R. Spivak, Kurunthachalam Kannan

**Affiliations:** 1 Department of Community and Environmental Health, Boise State University, Boise, ID USA; 2 School of Public Service, Boise State University, Boise, ID USA; 3 Department of Sociology, Boise State University, Boise, ID USA; 4 Center for Excellence in Environmental Health and Safety, Boise State University, Boise, ID USA; 5 Department of Pediatrics, New York University School of Medicine, New York, NY 10016 USA

**Keywords:** Agricultural workers, Women, Latina, Pesticides, Urinary biomonitoring

## Abstract

**Background:**

Women who work in agriculture may have greater risk of pesticide exposure than men who share this occupation. Despite an increase in the fraction of the agricultural workforce comprised by women, few studies have characterized pesticide exposure in the USA with a focus on among these workers.

**Objective:**

This pilot study aimed to describe pesticide exposure in a cohort of Latina farmworkers in farming communities in southwestern Idaho.

**Methods:**

We collected urine samples from 29 Latina farmworkers, which were analyzed for 11 pesticide biomarkers. We evaluated the effect of pesticide spray season on urinary biomarker levels, and explored the effect of self-reported status as a pesticide handler on measured exposures.

**Results:**

No significant differences were found between biomarker levels in samples collected during the nonspray and spray seasons. We observed 11 extreme outlying values in samples collected during the pesticide spray season. The most extreme outlying values (MDA: 51.7 ng/mL; 3-PBA: 11.8 ng/mL; *trans*-DCCA: 23.4 ng/mL; and 2,4-D: 31.1 ng/mL) were all provided during the spray season by women who reported loading, mixing or applying pesticides.

**Conclusions:**

These results provide suggestive evidence that Latina farmworkers who handle pesticides during the spray season may be at an increased risk of exposure to organophosphate and pyrethroid insecticides, as well as the herbicide 2,4-D. We recommend that future research into pesticide exposures among farmworkers should include particular focus on this group.

## Introduction

Farmworkers may experience disparities related to health and well-being, as they face a unique intersection of risk factors that have been linked to adverse health outcomes. Social and cultural characteristics associated with their ethnicity, nationality, immigration status, social class, and rural location often occur alongside occupational hazards, including opportunities for injuries and potential exposures to pesticides, fertilizers, and other chemicals; diesel fuels and exhaust; ultraviolet radiation; biologically active dusts; and zoonotic viruses and bacteria [1–13].

Over the past several decades, there has been a marked increase in the percentage of farmworkers who are women, a phenomenon known as “the feminization of agriculture” [14]. The Food and Agriculture Organization of the United Nations reports that women comprise over 40% of the total agricultural workforce in the developing world: in the Near East and North Africa, the female share of the agricultural workforce rose from ~30% in 1980 to over 40% in 2011, and from ~45% to nearly 50% in East and Southeast Asia and sub-Saharan Africa over the same period [15]. This trend is not limited to the developing world. In the USA, the proportion of women in the agricultural workforce grew from 25% in 1989 to 32% in 2016 [16, 17]. In Idaho, estimates of the magnitude of these changes at the state and local levels are scarce, but our observations suggest a similar pattern. In a recent study among hops workers in southwestern Idaho, we estimated that more than 50% of the farm labor force was comprised of women [18].

Female farmworkers may be at greater risk for adverse health outcomes than their male counterparts, perhaps in part due to potential for increased occupational hazards such as pesticide exposures. Two US studies investigated the rate of acute pesticide poisonings among women and men working in agriculture between 1998 and 2005 [19] and 1998 and 2007 [20]. These studies employed data from the California Department of Pesticide Regulation and the Sentinel Event Notification System for Occupational Risks (SENSOR)-Pesticides Program and found a nearly twofold higher rate of acute pesticide poisoning among women working in agriculture compared to men. This was consistent with a study among pesticide applicators in southern China, which found the prevalence of acute pesticide poisoning to be twice as high in women as in men [21].

While the reason for these differences is not entirely clear, researchers have hypothesized that these findings may be due to differences in personal protective equipment (PPE) use and other protective activities between men and women in the agricultural workforce [19, 22]. In a study among Hmong farmers in Thailand, researchers reported that “Hmong women have less Thai language skills than men and less information concerning hazards of exposure or use of protective clothing” [22]. Another study among agricultural workers in the USA found that women were significantly less likely to wear PPE than men [19]. It is also possible that such differences could be due to a lack of availability of appropriate PPE, as opposed to simply knowledge or training. Research in the construction and mining industries has shown that female workers may have difficulty accessing properly fitting PPE, as it is typically designed for males, and this may be true in the agricultural industry as well [23–26]. Women and men may also work on different crops, resulting in differences in patterns of exposure and types of pesticides used [20]. Differences in agricultural duties between women and men may also lead to different levels of pesticide exposure. Women are less likely than men to operate machinery and thus may be more likely to have direct exposure to crops, including cutting, sorting, and harvesting [20, 27, 28].

Research also suggests that physiological differences between women and men may result in an increased susceptibility to the adverse health effects associated with pesticide exposures [29]. Female hormonal-related systems, mainly the reproductive system, may be adversely affected by pesticide exposure, and women may be more susceptible to pesticides during hormonal-based processes such as pregnancy, lactation, and menopause [29, 30]. There is also speculation, though inconclusive, that hormonal-related cancers in women, including breast, endometrium, ovary, bone, and thyroid, may be related to endocrine disrupting pesticides [29, 31]. In addition, previous studies have suggested that susceptibility to lipophilic pesticides may be increased in women due to higher levels of adipose tissue compared to men [29].

In sum, the past few decades have seen a marked increase in the number of women working in agriculture [15]. There is also evidence to suggest that the incidence of acute pesticide poisonings is higher in women farmworkers than men [19–22], and it is also possible that women farmworkers may be more susceptible to the adverse effects of pesticide exposure than men [29–31]. In this pilot study, we aim to characterize pesticide exposure via repeated urinary biomonitoring among a cohort of 29 women working in agriculture in southwestern Idaho in 2019, and to compare these exposure levels to those observed in other farmworking populations and in nationally representative samples.

## Methods

### Study participants

We recruited Latina agricultural workers who were specifically involved in fieldwork in southwestern Idaho, primarily from the farming communities of Nampa, Caldwell, Wilder, Marsing, Homedale, Parma, and Nyssa. Participant recruitment occurred between October 2018 and June 2019 at local community organizations including Migrant and Farmworker Head Start programs, and community events such as local festivals and health fairs. In order to enroll, women had to be aged 18 years or older, identify as Latina during the eligibility screening and informed consent process, and had to report employment as a farmworker during the previous year. We defined farmworker based on the United Farm Workers 2003 definition, which includes individuals working in any of the following agricultural sectors: cultivation and tilling of the soil; dairying; production, cultivation and growing; harvesting; raising of livestock, furbearing animals, and poultry; and practices incidental to farming including packinghouse employees [32]. All study components were conducted in English or Spanish based on each participant’s language preference. This study was reviewed and approved by Boise State University Institutional Review Board.

### Survey data

All potential participants were asked verbally, in their preferred language, whether they were “a Latina farmworker over the age of 18, who has worked in Idaho agriculture over the past 12 months.” Answers were recorded, and those who responded “yes” were asked to complete a survey, which was based on several existing instruments, including a survey we previously developed to assess food quality and availability among Latina farmworkers [18], the National Agricultural Workplace Survey [33], and surveys of housing conditions and social isolation among farmworkers in the southeastern USA [34, 35]. The survey included six specific domains of inquiry: sociodemographics; food security and food provisioning; housing conditions; social isolation; access to medical care; and occupational hazards. Key sociodemographic and occupational hazard questions included participant’s history working in agriculture, sociodemographic characteristics including age, income, and whether they were best described as Mexican-American, Mexican, Chicana/o, Puerto Rican, other Hispanic, or not Hispanic or Latino/a, and occupational pesticide exposure.

Specific questions related to the potential for occupational pesticide exposure included whether or not participants had loaded, mixed, or applied pesticides in the past year (“yes/no/don’t know”), onto which crops the pesticides had been applied (“select all that apply: sugarbeets, onions, hops, peas, hay, mint, corn, grapes, potatoes, soy, barley, beef, dairy, other”), what specific types of pesticides had been applied (“select all that apply: insecticide, herbicide, fungicide, do not know”), and what equipment was used for application (“select all that apply: backpack sprayer, airblast sprayer, other”). They were also asked whether they had been given training or instructions in the safe use of pesticides (“through video, audio, cassette, classroom lectures, written material, informal talks, or by any other means”) during the past year with their current employer (“yes/no/don’t know”). Finally, as an indicator of compliance with the Worker Protection Standard (WPS), they were asked whether their current employer provided water to wash hands every day (“yes/no/don’t know”).

### Urinary biomonitoring

All participants who completed the survey were asked to take part in a urinary biomonitoring component of the study. For this component, each participant was asked to provide two urine samples for pesticide exposure analysis. We aimed to collect one urine sample during the time of year when pesticides are not typically applied in agriculture in Idaho (the “nonspray season”) and the other urine sample during active “spray season.”

For sample collection, each participant was provided with a prelabeled 4-oz polypropylene urine specimen collection cup and was asked to collect at least 10 mL of urine. Research staff recorded the date and time of sample receipt. Urine cups were placed into resealable plastic bags and transported on ice to our laboratory at Boise State University. Samples were kept in the laboratory refrigerator for no more than 48 h before processing. During processing, each sample was analyzed for color, clarity, and specific gravity via refractometry (Atago Urine Specific Gravity Refractometer, PAL 10-S), and aliquots were pipetted into 5-mL cryovials and stored at −80 °C.

### Sample analysis

Once all samples were collected, they were shipped overnight on dry ice in a single batch to the Wadsworth Center at the New York State Department of Health. The samples were analyzed for four specific metabolites of organophosphate (OP) insecticides: 2-isopropyl-4-methyl-6-hydroxypyrimidine (IMPY, a metabolite of diazinon), malathion dicarboxylic acid (MDA, a metabolite of malathion), *para*-nitrophenol (PNP, a metabolite of parathion and methyl parathion as well as other chemicals), and 3,5,6-trichloro-2-pyridinol (TCPY, a metabolite of chlorpyrifos and chlorpyrifos-methyl). Samples were also analyzed for five metabolites of pyrethroid insecticides: 3-phenoxybenzoic acid (3-PBA, a nonspecific metabolite of several pyrethroids including cyhalothrin, cypermethrin, deltamethrin, fenpropathrin, permethrin, and tralomethrin), 4-fluoro-3-phenoxybenzoic acid (4F-3-PBA, a metabolite of cyfluthrin and flumethrin), *trans*- and *cis*-isomers of 3-(2,2-dichlorovinyl)-2,2-dimethylcyclopropane carboxylic acid (*trans-*DCCA and *cis-DCCA*, metabolites of permethrin, cypermethrin, and cyfluthrin), and *cis*-3-(2,2-dibromovinyl)-2,2-dimethyl-cyclopropane-1-carboxylic acid (*cis*-DBCA, a metabolite of deltamethrin). Analysis also included two herbicides: 2,4-dichlorophenoxyacetic acid (2,4-D) and 2,4,5-trichlorophenoxyacetic acid (2,4,5-T).

Method details and quality control procedures for target analyte extraction have been previously described [36]. In brief, analytes were extracted using high-performance liquid chromatography (HPLC) and detected using tandem mass spectroscopy (MS/MS). Chromatographic separation was performed with a Waters ACQUITY Class I HPLC system, and the analysis was performed by Applied Biosystems API 5500 electrospray triple quadrupole mass spectrometer. The analysis controlled for sample-to-sample carryover of target analytes, instrumental drift in response factors, and contamination in laboratory materials and solvents. The trace concentrations of target analytes found in procedural blanks were subtracted from those measured in urine samples [36]. Limits of detection (LODs) were 0.005 μg/L (4F-3-PBA); 0.007 μg/L (IMPY); 0.010 μg/L (TCPY, MDA, 3-PBA, *trans*-DCCA, *cis*-DCCA, 2,4-D); 0.025 μg/L (PNP); and 0.050 μg/L (*cis*-DBCA, 2,4,5-T).

In order to assure accuracy and reliability through quality assurance (QA) checks, we also randomly selected six urine samples (14% of the total sample size) as duplicates for laboratory analysis. The laboratory analysts were blinded to the inclusion of these samples, which were shipped in the same batch with the primary samples.

### Data analysis

For pesticide biomarker analysis, we first conducted QA checks by calculating the average relative percent difference (RPD) between the six pairs of duplicate samples for each biomarker. The average RPD ranged from 7% (PNP) to 82% (*cis*-DCCA), although for the majority of biomarkers, the average RPD between duplicate pairs was ≤25%. We also calculated the detection frequency for each biomarker. Biomarkers with an average RPD < 40% among QA samples and frequency of detection > 50% among the sample set as a whole were included in our primary analyses.

Samples containing biomarker concentrations at levels below the laboratory LOD (<LOD) were substituted with LOD/$\sqrt 2$ [37]. Biomarker concentrations were adjusted for dilution based on specific gravity measurements, according to

$${C}_{{\mathrm{SG}} = }{C} \times \frac{{1.023 - 1}}{{{\mathrm{SG}} - 1}}$$

where *C*_SG_ is the adjusted result (ng/mL), *C* is the original measured concentration (ng/mL), 1.023 is the mean specific gravity for the full urinary biomonitoring dataset, and SG is the specific gravity of the individual sample [38].

We calculated the arithmetic mean, geometric mean (GM), the 50th, 75th, 90th, and 95th percentiles, and the maximum value for all biomarkers. We defined outliers as sample concentrations greater than or equal to 1.5 times the interquartile range (IQR) above the third quartile (Q3), or 1.5 times the IQR below the first quartile (Q1). “Extreme” outliers were defined as three times the IQR above Q3 or three times the IQR below Q1 [39].

We evaluated the potential relationship between time of collection (nonspray season/spray season) and biomarker concentrations. A sample was considered to be collected during the “nonspray season” if it was provided between January 1 and April 14, 2019, and during the “spray season” if was provided between April 15 and June 30, 2019, based on the period of active pesticide spray season in southwestern Idaho. If an individual participant contributed more than one sample during the same season, only one such sample was used (i.e., if a participant provided two samples after April 14, 2019, then the earlier of the two samples was excluded and the later of the two samples was included as part of the spray season dataset). The data were not normally distributed, and we therefore employed the nonparametric Mann–Whitney *U* test to compare pesticide exposure between samples collected during the nonspray season and the spray season.

We also explored the effect of pesticide handler status on biomarker concentrations. A participant was considered a pesticide handler if she selected “yes” when asked on the survey whether or not she had loaded, mixed, or applied pesticides in the past year. This analysis was considered exploratory due to sample size limitations.

Quantitative analysis, including descriptive statistics, frequencies, and measures of central tendency, was completed with SAS 9.4, Cary, NC.

## Results

### Study participants and survey data

A total of 29 women enrolled in this study. Among these 29 women, 14 (48%) women provided one urine sample and 15 (52%) women provided two urine samples, for a total of 44 samples.

The demographic characteristics are shown in Table 1. The average age was 39.5 ± 10.6 years. The majority of study participants identified as Mexican. For the majority of women, annual household income was between $20,000 and $49,999 for a household size of 3–5 people. Women had worked in agriculture for an average of 11 years, and reported working in agriculture an average of 7 months during the past year.

**Table 1:** Participant characteristics.

Variable	*N* (%) or sample mean (SD)
Age (years) (mean, SD)^a^	39.5 (10.6)
Race^b^	
White	7 (24%)
Non-White	10 (35%)
Missing	12 (41%)
Ethnicity^b^	
Mexican-American	3 (10%)
Mexican	26 (90%)
Chicana	1 (3%)
Other	0 (0%)
Missing	1 (3%)
Preferred language	
English	4 (14%)
Spanish	25 (86%)
Income (household, annually)	
Less than $10,000	8 (28%)
$20,000–$49,999	15 (52%)
$50,000 or more	1 (3%)
Missing	5 (17%)
Total number of people living in houshold (mean, SD)	5 (2)
Number of children living in household (mean, SD)	3 (1)
Total years worked in agriculture (mean, SD)	11 (11)
Months worked in agriculture (in past year) (mean, SD)	7 (4)

^a^One participant was excluded from analysis (improbable value, reported 2018 as year of birth).

^b^Participants could select more than one answer.

Occupational characteristics are shown in Table 2. The majority of participants reported working in agriculture in the spring and summer, and mostly with onions, corn, and potatoes. Despite WPS regulations requiring agricultural employers to provide yearly pesticide training to all workers [40], nearly one in five women reported that they did not receive such training. Approximately 25% of the women also reported that their employer did not provide water for handwashing every day.

**Table 2:** Occupational characteristics.

Variable	*N* (%)
Number (%) of women who report working in agriculture during each season^a,b^	
Spring	21 (72%)
Summer	21 (72%)
Fall	15 (52%)
Winter	11 (38%)
Number (%) of women who report working with each of the following crops^a,c^	
Onion	21 (72%)
Corn	17 (59%)
Potatoes	9 (31%)
Hops	7 (24%)
Grapes	5 (17%)
Mint	5 (17%)
Sugarbeets	3 (10%)
Employer provides water to wash hands every day	
Yes	21 (72%)
No	6 (21%)
Do not know	0 (0%)
Missing	2 (7%)
Received pesticide training from current employer (in past year)	
Yes	22 (76%)
No	5 (18%)
Do not know	1 (3%)
Missing	1 (3%)
Loaded, mixed, or applied pesticides (in past year)	
Yes	5 (18%)
No	22 (76%)
Do not know	1 (3%)
Missing	1 (3%)
Pesticide application equipment^a^	
Backpack sprayer	2 (7%)
Other (tank, tractor)	3 (10%)
Not applicable	24 (83%)
Type of pesticide applied^a^	
Insecticide	2 (7%)
Herbicide	3 (10%)
Fungicide	0 (0%)
Do not know	2 (7%)
Not applicable	24 (83%)

^a^Participants could select more than one answer.

^b^Spring defined as March, April, and May; Summer defined as June, July, and August; Fall defined as September, October, and November; and Winter defined as December, January, and February.

^c^No participants reported working with soy or barley, fewer than five participants reported working with peas, hay, dairy, or beef.

Five women reported loading, mixing, or applying pesticides at work in the past year (17%). Three of these women reported using a tank or tractor for application, and two reported using a backpack sprayer for application. Two women reported applying insecticides, three women reported applying herbicides, and two women did not know the type of pesticides applied. No women reported applying fungicides.

### Pesticide biomarkers

The urinary biomonitoring results, including frequency of detection and detection limits, for the full urinary biomonitoring dataset for all pesticide biomarkers are shown in Table 3. Frequency of detection ranged from 5% (c*is-*DBCA) to 100% (MDA, PNP, 3-PBA, 2,4-D). Among OP metabolites, urine samples contained the highest concentrations of PNP and MDA (GMs: 0.65 ng/mL and 0.59 ng/mL, respectively); among pyrethroid metabolites, 3-PBA and *trans*-DCCA were found in the highest concentrations (GMs: 0.58 ng/mL and 0.26 ng/mL, respectively); and 2,4-D (GM: 0.35 ng/mL) was found in the highest concentrations among the herbicides. All of the biomarker distributions were right-skewed with a long tail.

**Table 3:** Summary of pesticide biomarker data.

					Percentiles				Maximum value^a,b^
	Frequency of detection (%)	Limit of detection^a^	Mean^a,b^	Geometric mean^a,b^	50th^a,b^	75th^a,b^	90th^a,b^	95th^a,b^	
Organophosphate metabolites									
IMPY	39%	0.007	0.050	0.013	<LOD	0.044	0.13	0.16	0.68
MDA	100%	0.010	2.25	0.59	0.66	1.88	3.21	4.30	51.71
PNP	100%	0.025	0.85	0.65	0.57	0.98	1.98	2.17	3.09
TCPY	91%	0.010	0.58	0.28	0.47	0.73	1.09	1.87	2.45
Pyrethroid metabolites									
3-PBA	100%	0.010	1.17	0.58	0.49	0.94	1.85	3.21	11.79
4F-3-PBA	57%	0.005	0.025	0.013	0.012	0.038	0.058	0.077	0.14
* trans*-DCCA	91%	0.010	1.36	0.26	0.25	0.61	1.87	3.75	23.42
* cis*-DCCA	93%	0.010	0.99	0.21	0.17	0.81	2.06	3.85	15.07
* cis*-DBCA	5%	0.050	0.048	0.041	<LOD	<LOD	0.10	0.10	0.19
Herbicides									
2,4-D	100%	0.010	1.17	0.35	0.30	0.49	1.07	1.55	31.11
2,4,5-T	30%	0.050	0.074	0.054	<LOD	0.080	0.23	0.24	0.35

^a^ng/mL.

^b^Adjusted for specific gravity.

Biomarkers for which the QA metrics were met (average RPD of duplicates < 40%) and that were detected in at least 50% of samples were included in the primary analyses. These included three OP metabolites (MDA, PNP, TCPY), two pyrethroid metabolites (3-PBA and *trans*-DCCA), and one herbicide (2,4-D).

During the nonspray season, 14 of the 29 women (48%) provided at least one urine sample (one woman provided two samples during this season, for a total of 15 samples collected during the nonspray season). During the spray season, 24 of the 29 women (83%) provided at least one urine sample (five women provided two urine samples during this season, for a total of 29 samples collected during the spray season). Analyses of the association between season and exposure was restricted to include one sample per woman per season: one sample each from 14 women during the nonspray season, and one sample each from 24 women during the spray season. Twenty-four women (83%) reported that they did not handle pesticides and five (17%) reported that they did handle pesticides.

The measurements for nonspray season and spray season for all biomarkers can be found in Supplementary Table 1. No significant differences were found between samples collected in the nonspray versus spray seasons for any of the biomarkers measured.

**Fig. 1:**
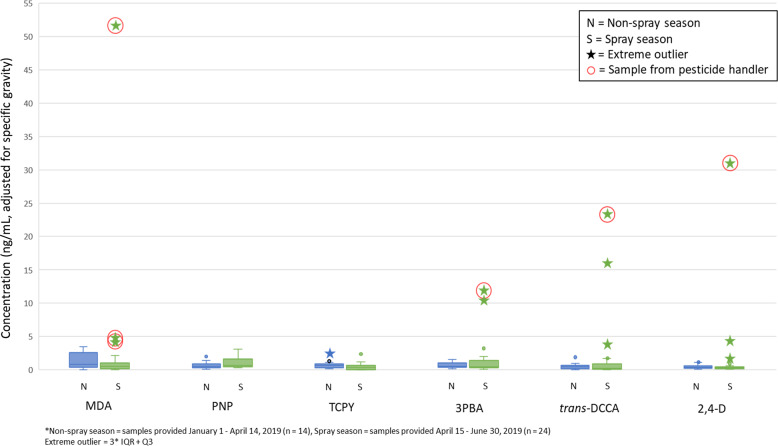
Key pesticide biomarker concentrations measured in urine samples collected from Latina farmworkers during the spray (S) and non-spray (N) seasons. Extreme outlying values are indicated with stars, and samples collected from pesticide handlers are circled.

While not statistically significant, an exploratory examination of the outliers suggested that some individuals might be more highly exposed than others, especially during the spray season. Specifically, we noted the number of “extreme” high outlying values in each season, as well as those that were from samples collected from pesticide handlers. The highest concentrations of five of the six primary biomarkers of interest were found in samples collected during the spray season: MDA (51.71 ng/mL), PNP (3.09 ng/mL), 3-PBA (11.79 ng/mL), *trans*-DCCA (23.42 ng/mL), and 2,4-D (31.11 ng/mL). As shown in Fig. 1, we observed five high outlying values (one of which was defined as “extreme”) during the nonspray season among all of the primary biomarkers. All of these outlying concentrations were below 5 ng/mL. For samples collected during the spray season, we observed 15 high outliers (11 of which were defined as “extreme”) among all of the primary biomarkers. Notably, the most “extreme” high outlying concentrations were measured in samples collected during the spray season from women who reported handling pesticides. We did not observe any low or “extreme” low outlying values for any biomarkers measured in any samples.

Among the OP metabolites, for PNP we observed one outlier in the nonspray season. For TCPY, we observed two outliers (one of which was defined as “extreme”) in the nonspray season and one outlier in the spray season. All of these outlying concentrations were below 5 ng/mL. For MDA, we observed three outlying value (all considered “extreme”). Notably, all of these outlying concentrations (4.30, 4.32, and 51.71 ng/mL) were measured in samples collected during the spray season and all were provided by women who reported handling pesticides.

With respect to the pyrethroid metabolites, no outlying values were measured for 3-PBA in the nonspray season, while three outliers (two of which were defined as “extreme”) were measured in samples collected during the spray season—the largest of which was provided by a pesticide handler (11.79 ng/mL). For *trans*-DCCA, we observed one outlying concentration in a sample collected during the nonspray season (1.87 ng/mL), and four that were collected during the spray season (three of which were defined as “extreme”). The most extreme of the spray season outliers was measured in a sample collected from a pesticide handler (23.42 ng/mL).

Among the herbicides, we observed one outlying concentration of 2,4-D measured in a sample collected during the nonspray season (1.07 ng/mL) and four outlying concentrations (three of which were defined as “extreme”) collected during the spray season, the largest of which was measured in a sample provided by a pesticide handler (31.11 ng/mL).

Two of the six women who reported that their employer did not provide water for handwashing every day also reported mixing, loading, or applying pesticides. In addition, one of these pesticide handlers who did not have access to water also reported that she did not receive any pesticide safety training. This same participant provided a urine sample during the spray season containing an “extreme” high outlying concentration of MDA.

## Discussion

This pilot study measured pesticide exposure among a small cohort of Latina farmworkers in southern Idaho. We did not observe any statistically significant differences in biomarker concentrations among samples collected during the nonspray season and spray season in our dataset as a whole. However, our exploratory analysis did suggest some trends in outlying results that may provide an important avenue for future studies. Specifically, these trends identify a subset of female agricultural workers who may be at an increased risk for pesticide exposure: women who handled pesticides during the spray season. These women provided samples that contained the most extreme outlying concentrations of MDA, 3-PBA, *trans*-DCCA, and 2,4-D. Though this study was limited by a small sample size, these preliminary results suggest that this particular group of agricultural workers may be at an increased risk of exposure to the OP malathion (the parent pesticide for MDA), certain pyrethroid insecticides (possibly permethrin or cypermethrin, parent pesticides for both 3-PBA and *trans*-DCCA [41]), and the herbicide 2,4-D. Though not as extreme as pesticide handlers, some women not handling pesticides during the spray season also provided samples that contained extreme outlying concentrations of 3-PBA, *trans*-DCCA, and 2,4-D. One possible explanation for this may be that nonhandlers could be exposed to pyrethroid insecticides and the herbicide 2,4-D, particularly during the spray season, possibly through field entry into areas treated with pesticides, contact with pesticide residues on soil or crops, or spray drift [42].

Few studies have investigated the risk factors associated with farm work specifically among women, making direct comparisons with our study results difficult. Data from the National Health and Nutrition Examination Survey (NHANES) on OP and pyrethroid pesticide exposure provide estimates for creatinine-adjusted urinary concentrations of MDA, PNP, TCPY, 3-PBA, *cis*-DCCA, *trans*-DCCA, *cis*-DBCA, and 4F-3-PBA stratified by sex and race/ethnicity in the US population as a whole [41, 43]. With respect to OP exposures, the 95th percentile concentration of IMPY, PNP, and TCPY was lower in our study than in women and Mexican-Americans in the NHANES population [43]. A study among pregnant women residing in the largely agricultural area of Salinas Valley, the Center for the Health Assessment of Mothers and Children of Salinas (CHAMACOS) study, measured pesticide metabolites from spot urine samples collected during two different prenatal interviews [44]. This study also detected a higher 95% percentile concentration of IMPY, PNP, and TCPY than we measured in our study [44]. However, the 95th percentile concentration of MDA in our study (4.30 ng/mL) was higher than among women in the NHANES population (2.1 ng/mL), Mexican-Americans in the NHANES population (1.7 ng/mL), and women in the CHAMACOS study (1.9 and 2.3 ng/mL) [43, 44]. The maximum MDA concentration in the CHAMACOS study was nearly identical to that found in a pesticide handler during the spray season in our study: 57.5 and 51.7 ng/mL, respectively [44].

In general, the detection frequencies for pyrethroid metabolites were similar between our study and NHANES: 3-PBA was the most frequently detected metabolite, while both 4F-3-PBA and *cis*-DBCA had low frequencies of detection [41]. The GM concentration for 3-PBA for women in our study (in the full urinary biomonitoring subset, and in both the nonspray and spray seasons: see Table 3 and Supplementary Table 1) was higher than females or Mexican-Americans in the USA (0.388 and 0.274 ng/mL, respectively) [41]. The 95th percentile concentration for *trans*-DCCA (in the full urinary biomonitoring subset and in the spray season: see Table 3 and Supplementary Table 1) was higher than females or Mexican-Americans in the USA (2.98 and 1.40 ng/mL, respectively) [41].

The levels of 3-PBA measured in samples collected from two women in our study during the spray season—a pesticide handler (11.79 ng/mL) and a nonhandler (10.55 ng/mL)—were notably higher than the 95th percentile concentration among women (4.43 ng/mL) and Mexican-Americans (1.18 ng/mL) in the NHANES population [41], as well as the 95th percentile concentrations among hired farmworker family households in the Mexican Immigration to California: Agricultural Safety and Acculturation Study (8.14 ng/mL, volume based) [45]. These same women also had notably higher *trans*-DCCA concentrations (handler: 23.42 ng/mL; nonhandler: 15.83 ng/mL) than the 95th percentile concentrations of females in the USA (2.98 ng/mL) and the 95th percentile of Mexican-Americans in the USA (1.40 ng/mL) [41].

With respect to herbicides, levels of 2,4-D were lower in our study than among females or Mexican-Americans in the 2009–2010 NHANES data [46]. The GM concentration of 2,4-D in our study was 0.35 ng/mL, compared to a GM concentration of 0.89 ng/mL among females in the US population and 1.24 ng/mL among Mexican-Americans in the USA [46]. While there have been several studies on 2,4-D exposure among farmworkers, to our knowledge, none have been specific to women. A 2005 study among 24 male farmers and 23 male non-farmers in Iowa found that the adjusted GM concentration of 2,4-D in urine collected from non-farmers (0.30 ng/mL) or from farmers who did not spray pesticides (0.54 ng/mL) was significantly lower than farmers who sprayed pesticides (11.0 ng/mL) [47]. Our data showed a similar pattern where the highest concentration of 2,4-D measured in any woman (31.11 ng/mL) was in sample collected from a pesticide handler during the spray season, while other extreme (but lower) outlying values of 2,4-D concentrations during the spray season were among nonhandlers.

In this exploratory analysis, season did not emerge as an important factor in pesticide exposure. We did not observe significant differences between any pesticide biomarkers for samples collected in the nonspray versus spray seasons. This is contrary to other studies which have shown pesticide exposure among farmworkers varies by time of year [48, 49]. This study collected one single spot urine sample per season, while other studies have collected multiple samples, which may explain this discrepancy. In addition, the majority of samples in this study were collected in the spring and early summer, as opposed to other studies that have collected urine samples throughout the entire summer.

This pilot study aimed to describe pesticide exposure among Latina farmworkers in southwest Idaho in order to inform hypotheses for future studies. While these results provide directions for future research, this study was clearly limited by a small sample size of 29 women and 44 urine samples, hindering the ability to fully explore potential differences pesticide exposure by time of collection and pesticide handler status. We aimed to collect two urine samples from all women completing the survey, but some women only provided one urine sample, and a small number of those providing two samples provided both samples during the same season. All urine was provided as individual spot samples, and were not collected as first morning voids. Due to the short half-lives of these biomarkers, these biomarkers do not reflect long-term exposures. The ability to collect multiple urine samples from each woman in each season would have provided us with a more reliable estimate of urinary biomarker concentrations.

In addition, there was potential for misclassification of participants by pesticide handler status, particularly for nonhandlers to be misclassified as handlers. The survey question used to determine pesticide handler status asked participants about loading, mixing, or applying pesticides during the past year, but participants that selected “yes” may not have been handling pesticides at the same time that they provided their urine samples. This study was also not able to evaluate other sources of pesticide exposure (e.g., residential use, dietary sources), and did not account for the possibility of pesticide exposure from occupational or residential pesticide use by other household members. This may be of particular importance for pyrethroid biomarkers, given that they are the most common residential insecticides used in the USA [41]. The sample of women for this study was based on convenience and not representative, and may not be generalizable to other agricultural populations given that we limited our study to Latina farmworkers specifically in southern Idaho. Finally, the distinction we chose between the “spray” and “nonspray” seasons was somewhat artificial, and we recognize that not every farm starts and stops spraying on the same day. However, the choice of dates for the season cutoff was based on detailed conversations with local experts on field preparation and pesticide application practices in this specific, fairly small, part of Idaho, and we believe that it is an accurate representation of regional practices in 2019. In addition, the very short biological half-life of the pesticides we measured in this study means that any changes in spraying practices would be reflected very quickly in urinary concentrations.

While preliminary data from this study suggest that female pesticide applicators working in the spray season may experience exposures to some pesticides that are higher than most of the general population, we recognize that more research is needed. This study provides insights into directions for future investigations in occupational pesticide exposure among female farmworkers, which will become increasingly important as the number of women working agricultural continues to grow [14]. This study also allowed our research group to strengthen valuable connections with a hard-to-reach, vulnerable farming population, which will enable future research.

In addition, WPS requirements regarding water for decontamination are not straightforward, but agricultural employers are required to provide all workers with supplies for routine and emergency decontamination, including “plenty of” soap and single use towels, and either one gallon of water per worker or three gallons of water per handler at the beginning of each work period [40]. The WPS also requires that employers provide annual pesticide safety training to all agricultural workers and pesticide handlers [40]. We found that approximately one-fourth of our study participants did not receive these regulatory protections.

While assessing the efficacy of pesticide safety training is challenging, some research does provide evidence for its value. A study among 82 farmers in northern Greece found that those who had received training on pesticide use (including use of spraying equipment, application parameters, use of PPE, and risks to human health and the environment) had more knowledge of pesticide uses and properties than those who had not received such training [50]. Farmers with such training also displayed increased safety behaviors such as wearing gloves when preparing spraying solutions and washing hands after application [50]. The importance of ensuring that all farm owners and labor contractors in the USA provide appropriate training and supplies to all workers is underscored by our finding that a pesticide handler who reported not having water for handwashing every day, and not receiving training on pesticides in the past year, provided a spray season urine sample with an “extreme” high outlying concentration of MDA.

In summary, this study found that individual Latina farmworkers in southern Idaho may have high occupational exposure to malathion, certain pyrethroid insecticides, and the herbicide 2,4-D. Future research should include particular focus on those Latina workers who handle pesticides during the spray season.

## Supplementary information

Supplemental Table 1
