# Supplementary material for: Measurement of urinary pesticide biomarkers among Latina farmworkers in southwestern Idaho
Source: J Expo Sci Environ Epidemiol. 2021 Jan 20;31(3):538–48. doi: 10.1038/s41370-020-00285-2 (PMC8134048; doi:10.1038/s41370-020-00285-2)
Supplement: Supplementary file 1 — Supplemental Table 1 [file 41370_2020_285_MOESM1_ESM.docx]

| **Supplemental Table 1. Pesticide Biomarker Data for Urine Samples Collected  During the Spray and Non-Spray Seasons⁺ (Non-Spray = 14 Samples, Spray = 24 Samples)** | | | | | | | | |
| --- | --- | --- | --- | --- | --- | --- | --- | --- |
|  | | | **Percentiles** | | | |  | |
|  | **Mean*** | **Geometric  Mean*** | **50th*** | **75th*** | **90th*** | **95th*** | **Maximum  Value*** | **p-valueˣ** |
| **Organophosphate Metabolites** | | | | | | | | |
| **IMPY** | | | | | | | | |
| Non-Spray Season | 0.058 | 0.016 | 0.0085 | 0.058 | 0.13 | 0.47 | 0.47 | 0.47 |
| Spray Season | 0.050 | 0.013 | <LOD | 0.039 | 0.067 | 0.16 | 0.68 |  |
| **MDA**** | | | | | | | | |
| Non-Spray Season | 1.32 | 0.70 | 0.82 | 2.47 | 3.21 | 3.46 | 3.46 | 0.28 |
| Spray Season | 3.02 | 0.52 | 0.50 | 0.044 | 4.30 | 4.32 | 51.71 |  |
| **PNP**** | | | | | | | | |
| Non-Spray Season | 0.68 | 0.52 | 0.55 | 0.83 | 1.42 | 1.98 | 1.98 | 0.32 |
| Spray Season | 0.99 | 0.75 | 0.65 | 1.53 | 2.17 | 2.48 | 3.09 |  |
| **TCPY**** | | | | | | | | |
| Non-Spray Season | 0.77 | 0.55 | 0.67 | 0.83 | 1.87 | 2.45 | 2.45 | 0.08 |
| Spray Season | 0.47 | 0.16 | 0.38 | 0.65 | 0.94 | 1.15 | 2.37 |  |
| **Pyrethroid Metabolites** | | | | | | | | |
| **3-PBA**** | | | | | | | | |
| Non-Spray Season | 0.69 | 0.58 | 0.54 | 0.95 | 1.33 | 1.54 | 1.54 | 0.59 |
| Spray Season | 1.58 | 0.62 | 0.46 | 1.19 | 3.21 | 10.55 | 11.79 |  |
| **4F-3PBA** | | | | | | | | |
| Non-Spray Season | 0.035 | 0.019 | 0.025 | 0.048 | 0.077 | 0.14 | 0.14 | 0.21 |
| Spray Season | 0.023 | 0.012 | 0.010 | 0.037 | 0.058 | 0.075 | 0.081 |  |
| ***trans*DCCA**** | | | | | | | | |
| Non-Spray Season | 0.50 | 0.29 | 0.43 | 0.62 | 0.96 | 1.87 | 1.87 | 0.41 |
| Spray Season | 2.08 | 0.25 | 0.22 | 0.70 | 3.75 | 15.83 | 23.42 |  |
| ***cis*DCCA** | | | | | | | | |
| Non-Spray Season | 0.53 | 0.13 | 0.12 | 0.45 | 1.60 | 3.85 | 3.85 | 0.35 |
| Spray Season | 1.33 | 0.24 | 0.19 | 0.99 | 2.06 | 7.63 | 15.07 |  |
| ***cis*DBCA** | | | | | | | | |
| Non-Spray Season | <LOD | <LOD | <LOD | <LOD | 0.074 | 0.10 | 0.10 | 0.66 |
| Spray Season | <LOD | <LOD | <LOD | <LOD | 0.10 | 0.10 | 0.16 |  |
| **Herbicides** | | | | | | | | |
| **2,4-D**** | | | | | | | | |
| Non-Spray Season | 0.44 | 0.36 | 0.39 | 0.58 | 0.84 | 1.07 | 1.07 | 0.30 |
| Spray Season | 1.79 | 0.37 | 0.28 | 0.43 | 1.55 | 4.25 | 31.11 |  |
| **2,4,5-T** | | | | | | | | |
| Non-Spray Season | 0.058 | <LOD | <LOD | 0.068 | 0.086 | 0.23 | 0.23 | 0.52 |
| Spray Season | 0.076 | 0.056 | <LOD | 0.092 | 0.16 | 0.24 | 0.35 |  |
| ⁺Non-spray season = samples provided January 1 - April 14, 2019, Spray season = samples provided April 15 - June 30, 2019 | | | | | | | | |
| *ng/mL, adjusted for specific gravity | | | | | | | | |
| ˣMann-Whitney U Test (2-sided) | | | | | | | | |
| **Included in primary analysis | | | | | | | | |
